# A comparison of integration methods for single‐cell RNA sequencing data and ATAC sequencing data

**DOI:** 10.1002/qub2.91

**Published:** 2025-02-27

**Authors:** Yulong Kan, Weihao Wang, Yunjing Qi, Zhongxiao Zhang, Xikeng Liang, Shuilin Jin

**Affiliations:** ^1^ School of Mathematics Harbin Institute of Technology Harbin China

**Keywords:** single‐cell omics, single‐cell integration, clustering analysis

## Abstract

Single‐cell genomics give us a new perspective to understand multivariate phenotypic and genetic effects at the cellular level. Recently, technologies have started measuring different modalities of individual cells, such as transcriptomes, epigenomes, metabolomes, and spatial profiling. However, integrating the results of multimodal single‐cell data to identify cell‐to‐cell correspondences remains a challenging task. Our viewpoint emphasizes the importance of data integration at a biologically relevant level of granularity. Furthermore, it is crucial to take into account the inherent discrepancies between different modalities in order to achieve a balance between biological discovery and noise removal. In this article, we give a systematic review for the most popular single‐cell integration methods and models involving cell label transfer, data visualization, and clustering task for downstream analysis. We further evaluate more than 10 popular integration methods on paired and unpaired gold standard datasets. Moreover, we discuss the data preferences of the limitations, applications, challenges and future directions of these methods.

## 1 INTRODUCTION

Single‐cell omics technologies are increasingly applied in many important fields, including biomedical, neuroscience, oncology, and microbiology, which provide high resolution insights into the complex gene regulation networks [1, 2, 3, 4] and different cellular processes [5, 6]. Methods for classifying cellular characteristics and processes at the single‐cell level are being increasingly applied to various molecular layers, including the genome (such as copy number variation and point mutations [7]), the epigenome chromatin accessibility [8], DNA methylation [9], histone modifications [10], RNA (such as RNA metabolism [11, 12], RNA isoforms [13]), and proteins [14, 15]. Moreover, there is ongoing research on developing experimental assays that can simultaneously capture two or three modalities within the same cell. Multimodal measurements, where different molecular features can be probed in the same cell, facilitate the development of innovative computational methods that analyze multimodal omics data. These measured data are called paired data. In the majority of cases, different modalities are not profiled from the same cells but from the same sample or tissue. These measured data are called unpaired data. Many sophisticated methods, such as the recently developed FIRM [16], Fugue [17], aka NIC [18], JSNMF [19], scIAE [20], GLOBE [21], DURIAN [22], and CLEAR [23] provide many flexible frameworks for joint modeling of variation across both modalities, technologies, and conditions.

Each of the biological insights provided by multimodal data help in the concomitant development of novel computational methods. For example, by conducting combined analysis, we can identify previously unrecognized cell populations [24] and facilitate the discovery of *cis*‐regulatory interactions [25, 26, 27, 28] or regulatory networks [25, 29, 30, 31, 32] specific to subpopulations. In addition to unraveling regulatory mechanisms in healthy cells, integrative multimodal analyses have the potential to unveil regulatory signatures [28] unique to cancer and provide insights into cancer evolution [33]. Ultimately, integrative analyses enable us to comprehend the interactions that occur within and between different molecular layers and their impact on gene expression. Thus, devising appropriate computational methods to integrate analyses of different data modalities is imperative [34, 35, 36]. One of the major challenges of multimodal sequencing is the heterogeneity across multiple datasets. Paired data analysis always regards cells as an anchor, which meets the obstacle of extreme data sparsity in joint profiling task. Unpaired data analysis aims to project multiple modalities into a common latent space or take advantage of transfer learning to fill the missing modalities. Deep learning is regarded as a breakthrough in artificial intelligence, which redefines our capabilities to analyze large‐scale single omics data by taking advantage of architectures of artificial neural networks [37, 38, 39]. Meanwhile, deep learning methods are versatile in many integration tasks compared with other methods [40].

**FIGURE 1:**
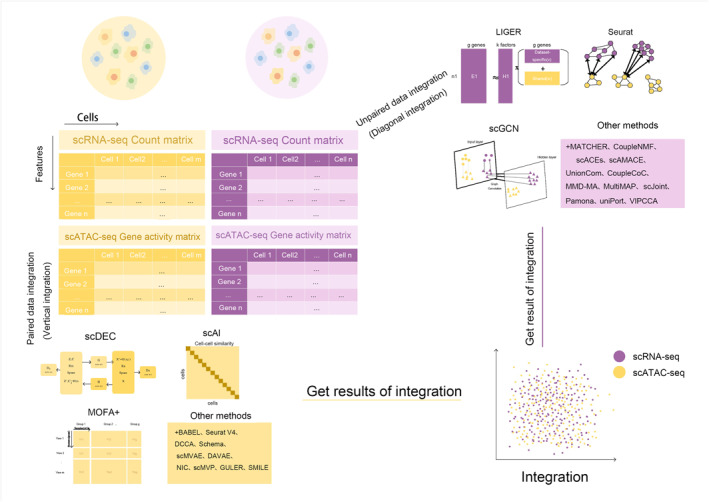
Workflow of typical single‐cell RNA sequencing and ATAC sequencing integration methods.

In this paper, we present a comprehensive review of the 16 recent advance methods for solving the challenge in integration of single‐cell RNA sequencing data and ATAC sequencing data. This review covers the classic and state‐of‐the‐art paired and unpaired integration methods. A workflow of integration analysis methods is provided (Figure 1). A summary of these methods and tools are provided in Tables 1, 2, 3, 4, 5, 6, including information of their language, access, modality technology, model, integration task, and scalability.

**TABLE 1:** Methods for paired data.

Algorithm	Language	Modality and technology
MOFA+ [41]	R	RNA expression + DNA methylation + chromatin accessibility (scNMT‐seq)
BABEL [42]	Python	RNA expression + chromatin accessibility (10× Genomics; SNARE‐seq; SHARE‐seq); RNA expression + protein epitope (CITE‐seq)
Seurat v4 [43]	R	RNA expression + protein epitope (CITE‐seq)
scAI [44]	MATLAB	RNA expression + chromatin accessibility (sci‐CAR);
	R	RNA expression + DNA methylation (scM&T‐seq)
DCCA [45]	Python	RNA expression + DNA methylation (scNMT‐seq); scATAC‐seq + scRNA‐seq (SNARE‐seq; SHARE‐seq; 10× Genomics)
scDEC [46]	Python	RNA expression + chromatin accessibility (10× Genomics)
Schema [47]	Python	RNA expression + chromatin accessibility (sci‐CAR; 10× Genomics)
scMVAE [48]	Python	RNA expression + chromatin accessibility (SNARE‐seq; scCAT‐seq)
DAVAE [49]	Python	RNA expression + chromatin accessibility (10× Genomics)
NIC [50]	MATLAB	RNA expression + chromatin accessibility
scMVP [50]	Python	RNA expression + chromatin accessibility (sci‐CAR; paired‐seq; SNARE‐seq; SHARE‐seq)
GULER [51]	Python	RNA expression + chromatin accessibility (sci‐CAR; SNARE‐seq; scCAT‐seq)
SMILE [52]	Python	RNA expression + chromatin accessibility (SNARE‐seq; sci‐CAR; SHARE‐seq); DNA methylation + chromosome structure data

*Note*: All information is from the original manuscript.

**TABLE 2:** Methods for unpaired data.

Alogorithm	Language	Modality and technology
MATCHER [53]	Python	RNA expression + chromatin accessibility; RNA expression + DNA methylation(scM&T‐seq; sc‐GEM); RNA expression + chromatin immunoprecipitation sequence
Seurat v3 [54]	R	RNA expression + chromatin accessibility (scRNA‐seq + scATAC‐seq; SMART‐seq2 + scATAC‐seq; SMART‐seq2 + sci‐ATAC‐seq1)
Liger [55]	R	RNA expression + DNA methylation;
CoupleNMF [31]	Python	RNA expression + chromatin accessibility (SMART‐seq + scATAC‐seq)
scACE [56]	R	RNA expression + chromatin accessibility
scAMACE [57]	R	RNA expression + chromatin accessibility
	Python	
scGCN [58]	Python	RNA expression + chromatin accessibility (sci‐CAR; 10× Genomics; scRNA‐seq + sci‐ATAC‐seq)
UnionCom [59]	Python	RNA expression + DNA methylation (sc‐GEM); RNA expression + DNA methylation + chromatin accessibility (scNMT‐seq)
CoupleCoC [60]	MATLAB	RNA expression + chromatin accessibility (scATAC‐seq + scRNA‐seq; sci‐ATAC‐seq + scRNA‐seq); RNA expression + DNA methylation (scRNA‐seq + snmC‐seq)
MMD‐MA [61]	Python	RNA expression + DNA methylation (scM&T‐seq)
MultiMAP [62]	Python	RNA expression + chromatin accessibility
scJoint [63]	Python	RNA expression + chromatin accessibility (scRNA‐seq + sci‐ATAC‐seq; scRNA‐seq + scATAC‐seq; SNARE‐seq); multimodal data (CITE‐seq + ASAP‐seq)
Pamona [64]	Python	RNA expression + DNA methylation (sc‐GEM); RNA expression + DNA methylation + chromatin accessibility (scNMT‐seq); RNA expression + chromatin accessibility (SNARE‐seq; 10× Genomics)
uniPort [65]	Python	RNA expression + chromatin accessibility (scRNA‐seq + scATAC‐seq)
VIPCCA [66]	Python	RNA expression + chromatin accessibility + DNA methylation (scNMT‐seq; scRNA‐seq + sci‐ATAC‐seq + sc‐methylation data)

*Note*: All information is from the original manuscript. Here we want to emphasize the sequencing technology of some modality data; the same below.

**TABLE 3:** Methods for paired and unpaired data commonly.

Alogorithm	Language	Modality and technology
Cobolt [67]	Python	RNA expression and chromatin accessibility (SNARE‐seq; 10× Genomics; SNARE‐seq + scRNA‐seq + scATAC‐seq)
MultiVI [68]	Python	RNA expression + chromatin accessibility (10× Genomics + scRNA‐seq + scATAC‐seq)

*Note*: All information is from the original manuscript.

**TABLE 4:** Models characteristics for unpaired data.

Algorithm	Underlying model	Integration task	Comments			
			Scalability	GA	ST	Others
MATCHER [53]	Manifold alignment	Vertical; diagonal		✓		
Seurat v3 [54]	CCA	Horizontal; diagonal	1000000 cells/5.11 h (64 GB memory and 3.6 GHz Intel core i9 processor)	✓	✓	
Liger [55]	iNMF	Diagonal		✓	✓	
CoupleNMF [31]	NMF	Diagonal				
scACE [56]	Probability model	Diagonal				Unsupervised
scAMACE [57]	Probability model	Diagonal				Designed for three omics data
scGCN [58]	Graph convolutional model	Horizontal; vertical; diagonal	1000000 cells/9.08 h (64 GB memory and 3.6 GHz Intel core i9 processor)			Semi‐supervised; integration across species
UnionCom [59]	Manifold alignment	Horizontal; vertical; diagonal				Unsupervised; can be extended to be more than two modalities
CoupleCoC [60]	Transfer learning	Diagonal		✓		Unsupervised; integration across species
MMD‐MA [61]	Manifold alignment	Vertical; diagonal				Unsupervised
MultiMAP [62]	Manifold alignment	Horizontal; vertical; diagonal	600000 cells/1.06 h (3.1 GHz Intel i7 core and 218 GB RAM)	✓	✓	
scJoint [63]	Transfer learning + NN	Vertical; diagonal	1000000 cells/2 h (dual 40 cores, 768 GB memory Intel(R) Xeon(R) gold 6148 processor; dual RTX2080TI graphics processing units)	✓		Semi‐supervised; integration multi‐modality profile
Pamona [64]	Manifold alignment	Vertical; diagonal				
uniPort [65]	VAE	Horizontal; vertical; diagonal	320000 cells/0.18 h (AMP EPYC 7302 16‐Core Processor, 256 GB DDR4 memory and NVIDIA GPU Tesla T4)	✓	✓	
VIPCCA [66]	CCA + deep neural network	Horizontal; diagonal	✓			

Abbreviations: CCA, canonical correspondence analysis; GA, the activity matrix of ATAC; iNMF, integrative non‐negative matrix factorization; NMF, non‐negative matrix factorization; NN, neural networks; ST, spatial transcriptomics; VAE, variational autoencoder.

**TABLE 5:** Models characteristics for paired data.

Algorithm	Underlying model	Integration task	Comments			
			Scalability	GA^a^	ST^b^	Others
MOFA+ [41]	PCA + factor analysis	Horizontal; vertical				Unsupervised; can be extended to be more than two modalities
BABEL [42]	AE	Vertical; mosaic				
Seurat v4 [43]	WNN	Horizontal; vertical		✓	✓	Unsupervised
scAI [44]	Matrix factorization	Vertical				Unsupervised
DCCA [45]	VAE‐attention transfer	Vertical; mosaic				Unsupervised
scDEC [46]	GAN	Vertical; horizontal;				Unsupervised
Schema [47]	Metric learning	Vertical			✓	Supervised; can be extended to be more than two modalities
scMVAE [48]	GMM‐VAE	Vertical		✓		Unsupervised
DAVAE [49]	VAE + domain adversial	horizontal; vertical; diagonal		✓	✓	Unsupervised
NIC [50]	Joint NMF	Vertical				
scMVP [50]	GMM‐VAE	Vertical	100000 cells/0.83 h (10 core Intel Xeon E5‐2680 with 32 GB RAM and NVDIA 1080 TI GPU with 1 GB RAM)			
GULER [51]	jointNMF + NN	Vertical		✓	✓	
SMILE [52]	AE	Horizontal; vertical		✓		Can be extended to be more than vertical two modalities

Abbreviations: AE, autoencoder; GA, the activity matrix of ATAC; GAN, generative adaversarial network; GMM, Gaussian mixture model; NMF, non‐negative matrix factorization; PCA, principal component analysis; ST, spatial transcriptomics; VAE, variational autoencoder; WNN, weighted nearest neighbor.

^a^ Whether the scATAC‐seq data need to be converted into the form of gene activity matrix in this algorithm.

^b^ Whether the algorithm integrates transcriptome and spatial resolve data.

**TABLE 6:** Models characteristics for paired and unpaired data commonly.

Algorithm	Underlying model	Integration task	Comments			
			Scalability	GA^a^	ST^b^	Others
Cobolt [67]	VAE + domain adversial	Vertical; diagonal				
MultiVI [68]	VAE	Vertical; diagonal; mosaic				Supervised

Abbreviations: GA, the activity matrix of ATAC; ST, spatial transcriptomics; VAE, variational autoencoder.

^a^ Whether the scATAC‐seq data need to be converted into the form of gene activity matrix in this algorithm.

^b^ Whether the algorithm integrates transcriptome and spatial resolve data.

## 2 STATISTICAL CHALLENGES ASSOCIATED WITH SINGLE‐CELL PAIRED AND UNPAIRED MULTIMODAL DATA INTEGRATION

Due to the wide range of experimental assays, modalities, and biological questions the task of integrating multimodal data is not a singular well‐defined task. We can distinguish between two distinct integration tasks: integrating data from multiple modalities that originate from the same cell (referred to as paired data), and integrating data from different cells that belong to similar but nonidentical cell populations (referred to as unpaired data).

In the case of paired data, where cell‐to‐cell correspondences are known, the primary objective is to improve the identification of cell states. On the other hand, unpaired data integration primarily focuses on identifying cell‐to‐cell correspondences. These fundamental differences in integration tasks have led to the development of tools that are generally designed for either paired or unpaired data integration.

- Heterogeneous data modalities: Molecular readouts obtained using different assays often exhibit distinct statistical properties and require specialized methods with different statistical assumptions.
- Overfitting and scalability: As the number of molecular layers and features increases, modeling strategies face the risk of overfitting if not properly regularized.
- Assay noise and missing data: Due to the small amounts of starting material, single‐cell technologies inherently introduce noise resulting in substantial technical noise.
- Biological factors variation between modalities: Variations can arise due to chromatin priming, where changes in chromatin accessibility precede gene expression changes or convergence of lineages.

## 3 INTEGRATION METHODS

We introduce the data integration technologies and methods for linking different datasets. Meanwhile, we focus on the methods developed for paired data and unpaired data. Paired data integration can also be regarded as vertical integration with cells as the anchors, whereas unpaired data integration can be regarded as diagonal integration with no anchors in a high‐dimensional space, and a few methods for paired and unpaired data commonly. In this section, we review the motivation, characteristics, innovation points, applicable fields, limitations, and differences between these data integration methods.

### 3.1 Methods for paired data

There are a growing number of platforms that allow for measuring several modalities on single cells. Many developed technologies enable simultaneously measurement of different omics data on the same cells. To leverage the information implied in multiomics data, different kinds of computational methodologies for integrative analysis of paired high‐dimensional multimodality data are proposed. The brief summary is shown in Table 1.

#### 3.1.1 MOFA+

Argelaguet et al. developed a statistical framework for comprehensive integration of multimodal single‐cell data [41].

Motivation and innovation points: MOFA+ innovatively combines factor analysis with multiomics data integration, allowing the decomposition of covariation across different omics layers into latent factors. It also handles heterogeneity across samples and conditions.

Applicable fields: MOFA+ is applicable to various fields involving multiomics data integration, such as cancer research, developmental biology, and precision medicine.

Limitations: MOFA+ requires careful handling of missing data and assumes linear relationships between the omics layers, which may limit its applicability to datasets with nonlinear relationships.

#### 3.1.2 BABEL

Wu et al. designed BABEL, a method that enables cross modality translation between single‐cell multiomics profiles thus enabling multiomics analysis [42].

Motivation and innovation points: BABEL innovatively employs Bayesian latent embedding techniques to align and integrate single‐cell data from different research institutions or laboratories. It enables the comparison and harmonization of datasets from diverse sources.

Applicable fields: BABEL is particularly useful in large‐scale collaborative studies, biobanks, and multi‐center research projects involving single‐cell data integration.

Limitations: BABEL’s performance may be influenced by batch effects and dataset size variations. It requires a sufficient number of shared cell types between datasets for accurate alignment.

#### 3.1.3 Seurat v4

Seurat v4 presented by Hao et al. is a weighted nearest neighbor analysis framework to integrate paired multiomics data [43].

Motivation and innovation points: Seurat v4 introduces novel integration techniques, including the “anchors” approach, to align and integrate multiomics data. It provides comprehensive functionalities for data preprocessing, integration, and downstream analysis.

Applicable fields: Seurat v4 is widely applicable across various research fields involving single‐cell multiomics data, such as immunology, neurobiology, and developmental biology.

Limitations: Seurat v4’s integration performance can be affected by batch effects and the presence of rare cell types. It may also be computationally demanding for large‐scale datasets.

#### 3.1.4 scAI

An unsupervised single‐cell integration and aggregation approach, scAI, was presented by Jin et al. to deconvolute cellular heterogeneity from paired multiomics data [44].

Motivation and innovation points: scAI leverages deep learning algorithms to learn the mapping relationships between different single‐cell datasets, enabling robust integration and analysis. It can handle complex and high‐dimensional data.

Applicable fields: scAI is applicable to diverse fields that require the integration of single‐cell data, such as disease research, cell type classification, and drug discovery.

Limitations: scAI’s performance heavily relies on the availability of labeled training data and can be affected by dataset heterogeneity and batch effects. It may require substantial computational resources and expertise in deep learning.

#### 3.1.5 DCCA

By combining variational autoencoders (VAE) and the attention transfer model, Zou et al. proposed a deep cycle attention model for single‐cell multiomics data integrative analysis [45].

Motivation and innovation points: DCCA uses deep learning models to capture nonlinear relationships and identify the maximum correlation between multiomics datasets. It provides a low‐dimensional shared representation for integration and analysis.

Applicable fields: DCCA is applicable to various fields involving single‐cell multiomics data, such as cancer genomics, immunology, and systems biology.

Limitations: DCCA’s performance may be influenced by dataset size, noise, and nonlinear relationships. It requires careful parameter tuning and can be computationally intensive.

#### 3.1.6 scDEC

Inspired by the generative adversarial networks model, Liu et al. proposed scDEC for simultaneously learning the deep embedding and clustering of cells [46].

Motivation and innovation points: scDEC combines differential expression and clustering analysis to identify differences between cell types and integrate multiple single‐cell RNA sequencing datasets. It provides a unified framework for analysis and integration.

Applicable fields: scDEC is applicable to fields requiring the identification of cell type‐specific gene expression changes and the integration of single‐cell RNA sequencing data from different experiments.

Limitations: scDEC may be sensitive to noise and batch effects. It requires careful selection of parameters and assumes the availability of cell type annotations for training.

#### 3.1.7 Schema

Drew inspiration from metric learning, Singh et al. developed Schema for synthesizing single‐cell multi‐modalities data [47].

Motivation and innovation points: Schema provides functionalities for data preprocessing, cell type identification, and data visualization in the context of single‐cell multiomics data integration. It offers a flexible and user‐friendly framework for analysis.

Applicable fields: Schema is applicable to various fields that involve the integration and analysis of single‐cell multiomics data, such as developmental biology, stem cell research, and disease modeling.

Limitations: Schema’s performance depends on the quality of input data and may be affected by batch effects and technical variability. It requires careful parameter selection and may not handle extremely large datasets efficiently.

#### 3.1.8 scMVAE

Zou et al. presented a single‐cell multimodal VAE model [48], scMVAE, where each omics data was modeled as having one zero‐inflated negative binomial distribution.

Motivation and innovation points: scMVAE innovatively employs a multiview variational autoencoder framework to integrate and analyze single‐cell data from multiple modalities or omics layers. It captures the shared and specific features across modalities, enabling comprehensive multiomics analysis.

Applicable fields: scMVAE is applicable to various research fields that involve multi‐modal single‐cell data, such as cancer genomics, immunology, and developmental biology.

Limitations: The performance of scMVAE may be influenced by dataset heterogeneity, missing data, and the choice of hyperparameters. It requires careful validation and interpretation of the learned latent representation.

#### 3.1.9 DAVAE

Aim at integration for paired data of chromatin accessibility profile and transcriptome profile that are simultaneously measured from a same cell, Hu et al. proposed DAVAE [49].

Motivation and innovation points: DAVAE leverages adversarial training techniques in a deep variational autoencoder framework to integrate and analyze single‐cell data. It aims to capture the underlying data distribution, learn disentangled representations, and handle batch effects and dataset heterogeneity.

Applicable fields: DAVAE is applicable to diverse research fields involving single‐cell data integration and analysis, such as cell type identification, trajectory inference, and disease classification.

Limitations: DAVAE’s performance may be affected by the presence of rare cell types, noise, and imbalanced datasets. It requires careful parameter tuning and validation for different applications.

#### 3.1.10 NIC

NIC is a network‐based integrative clustering algorithm developed by Wu et al. [50].

Motivation and innovation points: NIC innovatively integrates multiomics data by incorporating prior knowledge from biological networks. It leverages network‐guided regularization to improve the integration accuracy and interpretability, enabling the discovery of biologically relevant patterns.

Applicable fields: NIC is particularly useful in research fields that involve biological network analysis and multiomics data integration, such as systems biology, disease mechanisms, and drug discovery.

Limitations: NIC’s performance may depend on the quality and completeness of the underlying biological networks. It may also be sensitive to the choice of network regularization parameters and the availability of prior knowledge.

#### 3.1.11 scMVP

Li et al. proposed a multimodal deep generative model, scMVP [69]. scMVP automatically learns the common low‐dimensional latent representation for multiomics data through a clustering consistency constrained multiview VAE model.

Motivation and innovation points: scMVP introduces a variational Poisson framework for the integration and analysis of single‐cell multiomics data. It models the count‐based nature of omics data and captures the interdependence between modalities, enabling comprehensive multiomics analysis.

Applicable fields: scMVP is widely applicable across various research fields involving single‐cell multiomics data, such as developmental biology, cancer research, and immune profiling.

Limitations: scMVP’s performance may be influenced by the sparsity and noise inherent in count‐based data. It requires careful consideration of normalization methods and handling of zero‐inflation effects.

#### 3.1.12 GULER

GULER [51] was presented by Peng et al. It integrates the single‐cell omics via combining three computational techniques including joint NMF, MNN, and convolutional deep neural network.

Motivation and innovation points: GULER innovatively integrates multiomics data by leveraging graph‐based constraints on the latent representation. It utilizes a graph regularization term to capture the shared and specific information across omics layers, enabling effective multiomics analysis.

Applicable fields: GULER is applicable to various research fields involving multiomics data integration and analysis, such as cancer genomics, precision medicine, and systems biology.

Limitations: GULER’s performance may depend on the quality of the underlying graph structure and the choice of graph regularization parameters. It requires careful consideration of network construction and validation.

#### 3.1.13 SMILE

SMILE [52] is a deep learning model developed by Xu et al.

Motivation and innovation points: SMILE innovatively integrates multiomics data using a latent embedding approach. It leverages deep neural networks to learn a low‐dimensional latent space that captures the shared and specific features across modalities, enabling comprehensive multiomics analysis.

Applicable fields: SMILE is applicable to diverse research fields involving multimodal single‐cell data, such as developmental biology, disease research, and cellular heterogeneity analysis.

Limitations: SMILE’s performance may be influenced by the choice of hyperparameters, dataset heterogeneity, and the presence of rare cell types. It requires careful validation and interpretation of the learned latent representation.

### 3.2 Methods for unpaired data

Although there are many techniques that can measure the data of different omics in a cell at the same time, these techniques are usually difficult to implement and the cost is quite high. In most cases, different modalities are not profiled from the same cells, but from the same sample or tissue. So the analysis framework for unpaired data is necessary. The brief summary is shown in Table 2.

#### 3.2.1 MATCHER

Welch et al. proposed MATCHER [53] that uses manifold alignment to infer single cell multiomics profiles and embed data into 1D space.

Motivation and innovation points: MATCHER innovatively combines cell hashing and error robustness techniques to enable accurate and multiplexed transcriptome annotation. It allows the simultaneous identification of multiple RNA species within individual cells, providing enhanced transcriptomics profiling capabilities.

Applicable fields: MATCHER is applicable to various research fields involving single‐cell transcriptomics, such as cell lineage tracing, spatial transcriptomics, and immune profiling.

Limitations: MATCHER’s performance may be influenced by technical factors, such as cell hashing efficiency and sequencing depth. It also requires careful optimization of experimental protocols and validation of the identified RNA species.

#### 3.2.2 Seurat v3

Seurat is a popular method for comprehensive integration of single‐cell data proposed by Stuart et al. [54].

Motivation and innovation points: Seurat v3 introduces novel integration techniques, including the “anchors” approach, to align and integrate single‐cell RNA sequencing data. It provides comprehensive functionalities for data preprocessing, integration, and downstream analysis, along with improved scalability and speed compared to previous versions.

Applicable fields: Seurat v3 is widely applicable across various research fields involving single‐cell RNA sequencing data, such as immunology, neurobiology, and developmental biology.

Limitations: Seurat v3’s integration performance can be affected by batch effects and the presence of rare cell types. It may also be computationally demanding for large‐scale datasets.

#### 3.2.3 Liger

To delineate shared and dataset‐specific features of cells while integrating single‐cell multiomics data, Welch et al. developed Liger [55].

Motivation and innovation points: Liger introduces an integrative analysis framework that combines multiple single‐cell RNA sequencing datasets. It utilizes a network‐based approach to integrate and compare the datasets, enabling the identification of shared and distinct cell types across experiments.

Applicable fields: Liger is applicable to research fields involving the integration of multiple single‐cell RNA sequencing datasets, such as comparative analysis, disease subtyping, and cross‐study validation.

Limitations: Liger’s performance may be influenced by dataset heterogeneity, batch effects, and the choice of network construction parameters. It requires careful consideration of data preprocessing steps and validation of the identified cell types.

#### 3.2.4 coupleNMF

Duren et al. introduced couple non‐negative matrix factorizations (coupleNMF), a method focus on solving the couple clustering problem for unpaired scRNA‐seq and scATAC‐seq which is formulated as an optimization problem [31].

Motivation and innovation points: coupleNMF introduces a coupled matrix factorization approach to integrate and analyze multiomics single‐cell data. It aims to capture the shared and specific patterns across different omics layers, enabling comprehensive multiomic analysis.

Applicable fields: coupleNMF is applicable to various research fields involving multiomics single‐cell data, such as cancer genomics, molecular profiling, and cellular heterogeneity analysis.

Limitations: coupleNMF’s performance may be influenced by the choice of factorization parameters, dataset sparsity, and noise. It requires careful validation and interpretation of the identified patterns across omics layers.

#### 3.2.5 scACE

Considering model‐based method can quantify the uncertainty of clustering result, Lin et al. developed scACE, a model‐based method focus on mutiomics data coupled clustering problem [56].

Motivation and innovation points: scACE introduces a context‐specific expression analysis framework for single‐cell data. It leverages probabilistic modeling techniques to identify genes that are differentially expressed under specific cellular contexts, enabling the characterization of cell states and transitions.

Applicable fields: scACE is applicable to diverse research fields involving single‐cell transcriptomics, such as cell fate determination, cell type identification, and trajectory inference.

Limitations: scACE’s performance may be influenced by the choice of modeling assumptions, noise in the data, and the availability of context‐specific information. It requires careful consideration of statistical significance and validation of the identified context‐specific genes.

#### 3.2.6 scAMACE

Wang et al. extended scACE to scAMACE to integrate three omics data [57], including gene expression, chromatin accessibility, and methylation.

Motivation and innovation points: scAMACE introduces a mixture autoencoder framework for single‐cell data analysis. It combines unsupervised and supervised learning techniques to identify cell types, discover rare cell populations, and infer gene regulatory networks.

Applicable fields: scAMACE is applicable to various research fields involving single‐cell transcriptomics, such as cell type classification, cell state characterization, and regulatory network inference.

Limitations: scAMACE’s performance may depend on the availability of labeled training data and the choice of model parameters. It requires careful validation and interpretation of the inferred cell types and regulatory networks.

#### 3.2.7 scGCN

For effective knowledge represented as cell‐type labels transferring task across different datasets, Song et al. proposed a graph convolutional networks algorithm called scGCN where each cell is viewed as a node [58].

Motivation and innovation points: scGCN uses graph convolutional networks to analyze single‐cell data. It leverages the graph structure of cellular neighborhoods to capture local and global interactions, enabling the identification of cell types, trajectory inference, and gene regulatory network analysis.

Applicable fields: scGCN is applicable to diverse research fields involving single‐cell data analysis, such as developmental biology, immunology, and disease research.

Limitations: scGCN’s performance may be influenced by the quality of the underlying graph structure, dataset heterogeneity, and the choice of network architecture and hyperparameters. It requires careful consideration of graph construction methods and validation of the inferred biological relationships.

#### 3.2.8 UnionCom

Through the generalizing unsupervised manifold alignment algorithm, Cao et al. developed UnionCom for topological alignment of single‐cell multiomics data [59].

Motivation and innovation points: UnionCom introduces a unified matrix factorization framework to integrate multiview data. It aims to capture the shared and complementary information across different views, enabling comprehensive multiview analysis and data integration.

Applicable fields: UnionCom is applicable to various research fields involving multiview data integration, such as multiomics analysis, multimodal imaging, and social network analysis.

Limitations: UnionCom’s performance may be influenced by the heterogeneity and noise in the multiview data. It requires careful consideration of view weighting and validation of the integrated representation.

#### 3.2.9 coupleCoC

coupleCoC integrative analysis single‐cell multiomics data by a coupled coclustering‐based unsupervised transfer learning model [60].

Motivation and innovation points: coupleCoC introduces a coupled clustering approach for the integration and analysis of multiple single‐cell datasets. It aims to identify shared cell clusters and discover dataset‐specific clusters, enabling comparative analysis and cross‐dataset validation.

Applicable fields: coupleCoC is applicable to research fields involving the integration of multiple single‐cell datasets, such as cell type identification, differential expression analysis, and cross‐study validation.

Limitations: coupleCoC’s performance may be influenced by dataset heterogeneity, batch effects, and the choice of clustering parameters. It requires careful consideration of data normalization and validation of the identified cell clusters.

#### 3.2.10 MMD‐MA

MMD‐MA is a manifold alignment algorithm developed by Liu et al. to embed cells measurements of different modalities into a latent space [61].

Motivation and innovation points: MMD‐MA introduces a maximum mean discrepancy‐based alignment approach for multidomain single‐cell data integration. It aims to reduce domain‐specific differences and enable joint analysis across multiple datasets, facilitating cross‐domain comparisons and integration.

Applicable fields: MMD‐MA is applicable to research fields involving the integration of multidomain single‐cell data, such as multiomics analysis, spatial transcriptomics, and multimodal imaging.

Limitations: MMD‐MA’s performance may be influenced by the heterogeneity and noise in the multidomain data. It requires careful consideration of alignment parameters and validation of the integrated representation.

#### 3.2.11 MultiMAP

Motivated by manifold geometry, Jain et al. developed MultiMAP, an algorithm for the integration of multiple datasets with novel graph construction on the shared manifold, weights of the graph’s edges and the optimization of the embedding [62].

Motivation and innovation points: MultiMAP introduces a multiomic multistate analysis framework for population‐level single‐cell data. It enables the identification and characterization of distinct cellular states across multiple omics layers, facilitating the study of cell heterogeneity and population dynamics.

Applicable fields: MultiMAP is applicable to diverse research fields involving population‐level single‐cell data, such as developmental biology, disease progression, and cellular reprogramming.

Limitations: MultiMAP’s performance may be influenced by the choice of modeling assumptions, dataset heterogeneity, and the availability of multiomics measurements. It requires careful interpretation of the identified cellular states and dynamic trajectories.

#### 3.2.12 scJoint

Lin et al. proposed a transfer learning method to integrate scRNA‐seq and scATAC‐seq using a neural network approach [63].

Motivation and innovation points: scJoint introduces a probabilistic modeling framework for joint analysis of single‐cell RNA‐seq and ATAC‐seq data. It enables the integration and analysis of gene expression and chromatin accessibility profiles, facilitating the study of gene regulation and cell type‐specific regulatory elements.

Applicable fields: scJoint is applicable to research fields involving the integration of single‐cell transcriptomics and epigenomics data, such as developmental biology, cell fate determination, and gene regulatory network analysis.

Limitations: scJoint’s performance may be influenced by technical factors, such as data sparsity, batch effects, and the choice of model priors. It requires careful normalization and validation of the integrated gene expression and chromatin accessibility profiles.

#### 3.2.13 Pamona

Pamona, formulated the integrate single cell multiomics data task as a partial manifold alignment problem and can be solved by a partial Gromov–Wasserstein (GW) optimal transport framework [64], was proposed by Cao et al.

Motivation and innovation points: Pamona introduces a multiomics data integration method based on non‐negative matrix trifactorization. It enables the joint analysis of transcriptomics, epigenomics, and proteomics data, facilitating the identification of molecular interactions and regulatory mechanisms.

Applicable fields: Pamona is applicable to research fields involving multiomics data integration, such as systems biology, disease mechanisms, and biomarker discovery.

Limitations: Pamona’s performance may be influenced by the heterogeneity and noise in multiomics data. It requires careful consideration of missing data imputation and validation of the identified molecular interactions.

#### 3.2.14 uniPort

uniPort [65] is a unified single‐cell data integration framework introduced by Cao et al.

Motivation and innovation points: uniPort represents an innovative approach to unsupervised multiomic data integration using deep learning techniques. By learning low‐dimensional representations, it provides a powerful tool for exploring shared and specific features across multiple omics layers and extracting meaningful insights from complex biological datasets.

Applicable fields: uniPort is applicable to various research fields involving the integration of multiomics data, such as genomics, transcriptomics, proteomics, and metabolomics. It can be used for understanding complex biological processes, identifying molecular interactions, and discovering biomarkers.

Limitations: uniPort’s performance may be influenced by the heterogeneity and noise in multiomics data, as well as the complexity and dimensionality of the datasets. It requires careful validation and interpretation of the learned representations and downstream analysis results. Additionally, as an unsupervised method, uniPort may not explicitly capture specific biological outcomes or predictive features.

### 3.3 Methods for paired and unpaired data commonly

Except for the integration of paired data or unpaired data, some methods also focus on the comprehensive integration of multimodality data with single modality data collected from related biological systems. The brief summary is shown in Table 3.

#### 3.3.1 Cobolt

Inspired by the latent Dirichlet allocation, Gong et al. developed the Cobolt method, a coherent framework for an integrative analysis of multimodality data and single modality data commonly done by transfer learning [67].

Motivation and innovation points: Cobolt introduces a method that combines lineage tracing information with single‐cell RNA sequencing data. It enables the reconstruction of lineage trajectories and the characterization of gene expression dynamics along these trajectories, providing insights into cellular differentiation and developmental processes.

Applicable fields: Cobolt is applicable to research fields involving the study of cellular development, lineage tracing, and cell fate determination. It can be used to understand embryonic development, tissue regeneration, and disease progression.

Limitations: Cobolt’s performance may be influenced by the quality of lineage tracing data, the choice of trajectory inference algorithms, and the sparsity of single‐cell RNA sequencing data. It requires careful validation and interpretation of the reconstructed lineage trajectories and gene expression dynamics.

#### 3.3.2 MultiVI

Ashuach et al. presented a probabilistic model, MultiVI, to integrate multiomics data with single modality datasets [68].

Motivation and innovation points: MultiVI introduces a variational inference framework for the integration of multiomics data. It leverages deep generative models to learn joint representations of multiple omics layers, enabling comprehensive analysis and interpretation of multiomics datasets.

Applicable fields: MultiVI is applicable to diverse research fields involving multiomics data integration, such as cancer research, systems biology, and precision medicine. It can be used for identifying molecular interactions, discovering biomarkers, and understanding disease mechanisms.

Limitations: MultiVI’s performance may be influenced by the heterogeneity and noise in multiomics data, as well as the choice of model architectures and inference algorithms. It requires careful validation and interpretation of the learned joint representations and downstream analysis results.

## 4 MODEL CHARACTERISTICS

Here we briefly introduce the characteristics of each method, such as what underlying model is used and whether it has scalability. The results are shown in Table 4 for paired data methods, Table 5 for unpaired data methods and Table 6 for the method for both paired and unpaired data.

### 4.1 Gene activity matrix

The computational methods we discussed before generally adopt two ideas to integrate data, one is obtaining separate embeddings for each modality and the other is aligning features empirically before aligning cells. Considering scATAC‐seq, different methods adopt different forms of input data. Specifically, to achieve the later ideas, many methods employ “gene activity matrix” to integrate with expression data such as scMVAE and VIPCCA. Gene activity matrix is summarized form scATAC‐seq peak counts, utilizing observed reads at gene promoters and enhancers, representing a synthetic scRNA‐seq dataset to leverage for integration. In the low‐dimensional space, anchors for integration can be identified between gene activity data and expression data.

### 4.2 Scalability

With the growing of single‐cell multiomics data sizes, scalability becomes a properties that the researchers are concerned about. It is expected that an algorithm can be expanded to a large‐scale dataset and have good integration properties at the same time. That is, when facing large‐scale data, the algorithm occupies less running memory and takes less time. Some methods can be extended to a large‐scale dataset of the order of ten thousand cells, and some even have considerable efficiency in processing cells up to millions. We noted the relevant information of the previously mentioned methodologies that can process more than 100000 cells, and took the number of cells, running time, and processor parameters used by the proposers as the reference to measure scalability. It should be noted that these reference data are all from the original papers of these methods.

### 4.3 Model for data integration

More commonly published methods employ different underlying model to implement integrative tasks, including matrix factorization, deep learning models, and manifold alignment, and so on, each with its own advantages and limitations. Matrix factorization was successfully applied to a variety of frameworks owing to its simplicity and interpretability but the method’s performance is usually sensitive to gene selection [70]. Because of the high dimension of data, many researchers are employing a deep learning model. Most existing deep learning methods are widely used albeit at the cost of reduced interpretability. Additionally, manifold alignment approaches, which aimed to align embedded low‐dimensional manifolds, demonstrated promising results in multiomics integration tasks but they are limited because they require distributions to match globally and have high computational complexity. Apart from that, other methods, such as the CCA probability model, are also applied in integration tasks [71]. The underlying models of each method and algorithm are listed in Tables 1, 2, 3.

### 4.4 Integration scRNA‐seq with spatially resolved data

Our previous discussions focused on the integration of single‐cell multiomics cross modal data, mainly discussing the integration of transcriptome and epigenome. In fact, in order to understand the interplay between intrinsic and extrinsic factors that underlie cellular communication and organ function, integrating transcriptome and spatial transcriptome also becomes a research hotspot. The integration of scRNA‐seq and spatial transcriptomics can generate high‐resolution maps of cell subpopulations in tissues. Many methods have the ability to integrate transcriptome and spatial transcriptome [72, 73, 74] while realizing the integration of transcriptome and epigenome. Here, we note in the table for the reference of researchers but we will not introduce it in detail.

## 5 EXPERIMENTS

In this section, we evaluate several integration methods designed for paired and unpaired data on some benchmark datasets. For paired data, we choose scRNA‐seq and scATAC‐seq from mouse kidney cells and cell line mixture of SNARE‐seq. Mouse kidney data is downloaded from the GitHub of Tabula Muris. SNARE‐seq is downloaded from the GSE139369 cell line mixture of SNARE‐seq proposed by Jin [27]. For unpaired data, we choose single cell RNA‐seq and ATAC‐seq from the mouse atlas data subset by Lin et al. [64] and Human fetal atlas data is downloaded from GSE156793 and GSE149683.

**FIGURE 2:**
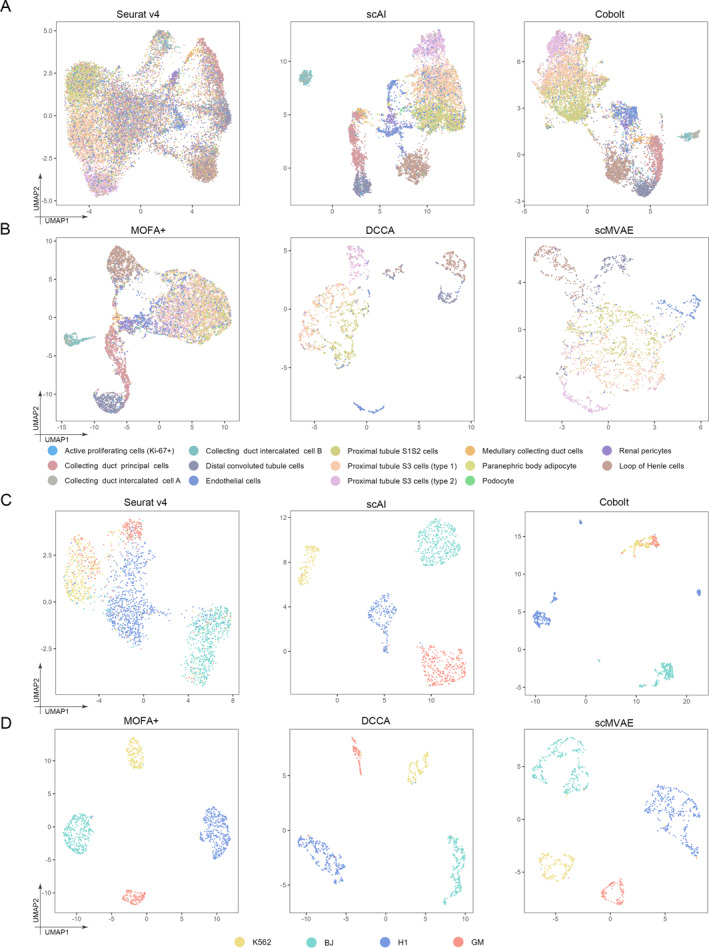
Integration of paired data. (A) UMAP visualization obtained from Seurat v4, scAI, and Cobolt colored by the true class. (B) UMAP visualization obtained from MOFA+, DCCA, and scMVAE colored by the true class. (C) UMAP visualization obtained from Seurat v4, scAI, and Cobolt colored by the true class. (D) UMAP visualization obtained from MOFA+, DCCA, and scMVAE colored by the true class.

**FIGURE 3:**
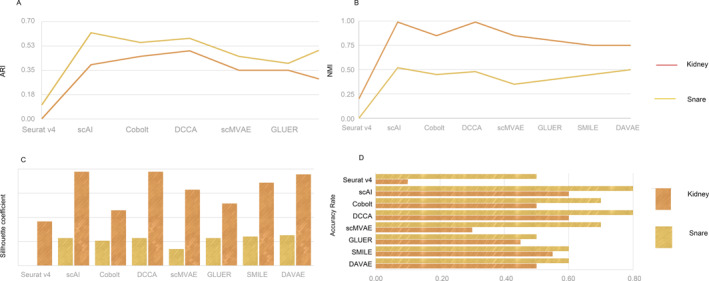
The quantitative assessments of paired methods and clustering performance comparison Seurat v4, scAI, Cobolt, DCCA, scMVAE, GLUER, SMILE, and DAVAE. The figure shows the clustering results of ARI (A), NMI (B), Silhouette coefficient (C), and the label transfer accuracy rate (D).

In the first experiment which targeted evaluating method for paired data, we analyzed and compared the efficiency of methods including Seurat v4, scAI, Cobolt, DCCA, scMVAE, GLUER, SMILE, and DVAE on paired data. Furthermore, the adjusted Rand index (ARI) [75], normalized mutual information (NMI) [76], silhouette coefficients [77], and transfer accuracy are used to evaluate the label migration effect of the algorithm. Here, we base the real cell type as classification. Transfer accuracy is the number of cells whose type is correctly predicted as a percentage of the total number of cells; the larger the value the better. Our results demonstrate that the performance of these methods is better in the snare dataset, but generally poor in the kidney dataset, this may be due to the fact that there are many cell subtypes in the kidney dataset, such as collecting duct intercalated cell A and collecting duct intercalated cell B, which also indicates that the algorithm does not perform well for datasets containing a large number of cell subtypes. Additionally, the performance of Seurat v4 is poorer than other methods, with class‐to‐class confusion, while the other methods are relatively good (Figure 2). For ARI and NMI metrics, scAI and DCCA showed advantages in both datasets. In the snare dataset, the ARI values of the two groups reached to 0.954 and 0.957, and the NMI values reached to 0.920 and 0.921, which indicated that the cluster tags assigned by them matched the real cell types very well (Figure 3A,B). scAI and DCCA also have higher silhouette coefficients, suggesting that the cell embedding they obtained better preserves the cell‐type signals (Figure 3C). Their transfer accuracy is relatively higher (Figure 3D). Seurat v4 provides a comprehensive analysis pipeline, whereas scAI utilizes deep learning techniques for tasks such as clustering and cell type annotation. Cobolt focuses on integrating and comparing multiple datasets, whereas DCCA captures correlations between datasets for integration. scMVAE and DVAE employ variational autoencoders for data integration and analysis. GLUER and SMILE also contribute to data integration and cell type comparison.

**FIGURE 4:**
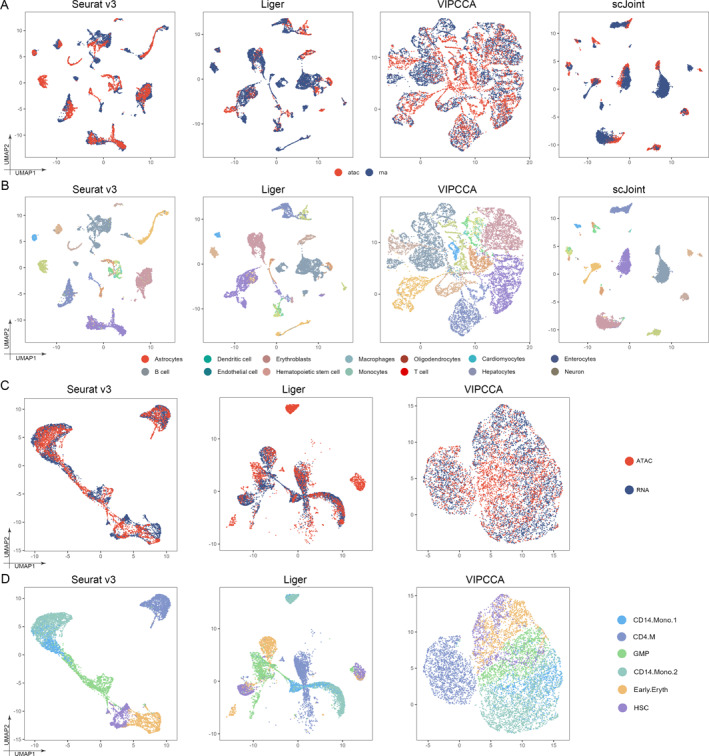
Integration of unpaired data. (A) UMAP visualization of mouse atlas data obtained from Seurat v3, Liger, VIPCCA, and scJoint of the subset colored by technology. (B) UMAP visualization of mouse atlas data obtained from Seurat v3, Liger, VIPCCA, and scJoint of the subset colored by the true celltype. (C) UMAP visualization of human fetal atlas data obtained from Seurat v3, Liger, and VIPCCA colored by technology. (D) UMAP visualization of human fetal atlas data obtained from Seurat v3, Liger, and VIPCCA colored by the true celltype.

**FIGURE 5:**
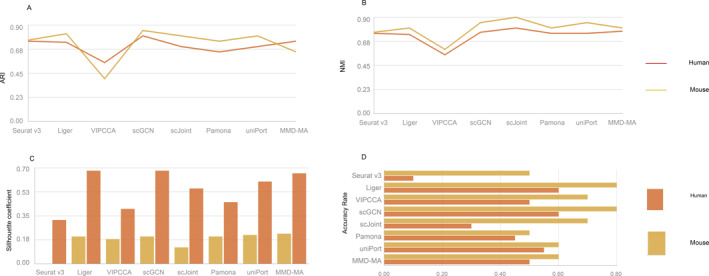
The quantitative assessments of unpaired methods and clustering performance comparison Seurat v3, Liger, VIPCCA, scGCN, scJoint, Pamona, and MMD‐MA. The figure shows the clustering results of ARI (A), NMI (B), Silhouette coefficient (C), and the label transfer accuracy rate (D).

For unpaired part, we conducted an integrated analysis of Seurat v3, Liger, VIPCCA, scJoint, scGCN, Pamona, uniPort, and MMD‐MA on unpaired data. Note that Liger is an unsupervised algorithm, and we manually assign labels to clusters. Our results show that Seurat v3, Liger, and scJoint effectively mixed different omics data, and effectively separated different types of cells. In addition, VIPCCA cannot tighten cells which belong to the same cell type, and the distance between cell clusters is so closer while others methods, especially scJoint, tend to have an excessive tightness for cells which belong to the same cell types and better separation of clusters with different cell types (Figure 4). For ARI metrics, NMI metrics and transfer accuracy, Seurat v3, Liger, scGCN, and scJoint all performed well, among which scGCN and scJoint were particularly outstanding, whereas VIPCCA was relatively inferior (Figure 5A–C). For silhouette coefficients, scGCN and scJoint performed better, whereas Seurat v3 and Liger performed worse. It can be seen that although all four methods can transfer cell labels relatively well, the cell embedding obtained using Seurat v3 and Liger does not retain the biological signal in the data very well (Figure 5D). These methods, including Seurat v3, Liger, VIPCCA, scJoint, scGCN, Pamona, uniPort, and MMD‐MA, have different characteristics that contribute to their respective clustering performance. Seurat v3 combines advanced statistical and machine learning techniques, providing a comprehensive analysis pipeline. Liger excels at integrating and comparing single‐cell data from different experiments or platforms by capturing shared cellular features. VIPCCA utilizes normalized linear models to minimize perturbations between datasets, enhancing data integration and clustering consistency. scJoint leverages deep generative models and variational inference to capture shared and specific information, facilitating integration across species or tissues. scGCN employs graph convolutional networks to model cell–cell interactions and local structures, improving clustering accuracy. Pamona integrates multiple datasets through joint dimensionality reduction and maximization of mutual information, resulting in consistent clustering results.

## 6 CONCLUSION

Rapid advances in single‐cell omics data have provided deep insight into prediction of gene expression dynamics and identification factors which drive cell fate. A variety of experimental technologies [24, 78, 79, 80] and different data modalities [34, 80, 81, 82, 83, 84] give us a new insight in identifying gene networks and molecular layers. The large amounts of scRNA‐seq data and scATAC‐seq data have facilitated the development of methods for integration of different modalities. Combined analysis of paired and unpaired single‐cell omics data open up new avenues to link multiple aspects of cellular identify. However, the heterogeneity of data modalities, the risk of overfitting, the absence of sequence reads, and the noise of sequence data offer more challenges for these analysis methods. In this review, we introduce paired and unpaired single‐cell integration strategies and discuss the limitations, challenges and opportunities of single‐cell integration methods.

For instance, many integration methods fail to account for dataset‐specific cell populations that may arise from technical variations between assays or biological distinctions across modalities. In the case of CCA‐based approaches [85, 86], the inherent assumption of CCA—that a linear relationship exists between variables—may be invalidated for gene expression and chromatin accessibility profiles, given the intricate nonlinear processes governing gene expression regulation. Methods relying on similarity kernels, which aim to discern a shared underlying manifold, might prove more suitable. However, the selection of a similarity kernel in these methods is intricate and dependent on the modality. Notably, the choice of the similarity kernel and the features used for its computation (e.g., genes or genomic regions) will impact the biological signals retrieved, akin to decisions regarding other preprocessing steps.

The increasing availability of atlas‐sized single‐cell omics datasets necessitates the development of methods capable of integrating expression and accessibility profiles for thousands to millions of cells [87, 88]. As the search space expands, finding appropriate cell‐to‐cell matches becomes more challenging. To address this, a constrained integration strategy has been proposed, wherein the alignment problem is divided into separate integration tasks for groups of cells that share a coarse‐grained cell identity (e.g., T cells and non‐T cells).

Moreover, most existing methods are not designed to handle the scale of thousands to millions of expression and accessibility profiles, often due to excessive runtime or memory requirements. Therefore, efforts are required to scale up existing approaches (as demonstrated with Seurat v3 in ArchR) and to develop inherently scalable methods, such as deep learning‐based approaches. It is crucial that these methods maintain scalability even when hyperparameter optimization is necessary. Hyperparameter optimization poses another bottleneck, particularly due to the reliance on labeled data. Paired multiomics data can contribute to addressing this challenge by providing labels for semi‐supervision and informing hyperparameter optimization strategies suitable for unpaired multiomics data integration tasks. We believe that, in the years to come, more versatile and powerful computational methods will efficiently and accurately harmonize a wide range of data and accelerate life sciences research [89, 90, 91].

## AUTHOR CONTRIBUTIONS

**Yulong Kan**: Writing—original draft; writing—review and editing. **Weihao Wang**: Data curation; formal analysis. **Yunjing Qi**: Validation; visualization. **Zhongxiao Zhang**: Data curation; resources; visualization. **Xikeng Liang**: Validation; visualization. **Shuilin Jin**: Supervision.

## CONFLICT OF INTEREST STATEMENT

The authors have no conflicts of interest to disclose.

## DATA AVAILABLE STATEMENT

Human SHARE‐seq (paired data) used in this study are available in the GEO database with accession ID GSE207308 Version:0.9 StartHTML:0000000105 EndHTML:0000001476 StartFragment:0000000141 EndFragment:0000001436. Mouse 10X Multiome kidney data used in this study could be downloaded from the homepage of 10× Genomics.

## ETHICS STATEMENT

All computational methods and results adhere to the principles of ethical research and transparency, ensuring that the data used in this study are handled responsibly and comply with institutional and international data protection regulations.
