# Correction to “A comparison of integration methods for single‐cell RNA sequencing data and ATAC sequencing data”

**DOI:** 10.1002/qub2.70023

**Published:** 2025-11-10

**Authors:** Yulong Kan, Weihao Wang, Yunjing Qi, Zhongxiao Zhang, Xikeng Liang, Shuilin Jin

**Affiliations:** ^1^ School of Mathematics Harbin Institute of Technology Harbin China

**Correction** to *Quantitative Biology*. 2025; e91. https://doi.org/10.1002/qub2.91.

The original version of this article unfortunately contained some mistakes.

(1) In the “Abstract” section, the text “However, integrating the results of multimodal single‐cell data to identify cell‐to‐cell correspondences remains a challenging task. Our viewpoint emphasizes the importance of data integration at a biologically relevant level of granularity. Furthermore, it is crucial to take into account the inherent discrepancies between different modalities in order to achieve a balance between biological discovery and noise removal.” was incorrect. This should have read: “Despite providing unprecedented insights into cellular heterogeneity, integrating multimodal single‐cell data to find cell‐to‐cell correspondences remains challenging, primarily due to the need for biologically granular integration and the management of technical and biological discrepancies between modalities.”

(2) In paragraph 1 of the “1 Introduction” section, the text “Methods for classifying cellular characteristics and processes at the single‐cell level are being increasingly applied to various molecular layers, including the genome (such as copy number variation and point mutations [7]), the epigenome chromatin accessibility [8], DNA methylation [9], histone modifications [10], RNA (such as RNA metabolism [11, 12], RNA isoforms [13]), and proteins [14, 15]. Moreover, there is ongoing research on developing experimental assays that can simultaneously capture two or three modalities within the same cell.” was incorrect. This should have read: “Single‐cell classification methods are now extensively applied across genomic [7], epigenomic [8], DNA methylation [9], histone modifications [10], transcriptomic [11–13], and proteomic [14–15] layers, with advancing research focused on multimodal assays that capture multiple molecular modalities within the same cell [16].”

(3) In paragraph 2 of the “1 Introduction” section, the text “For example, by conducting combined analysis, we can identify previously unrecognized cell populations [24] and facilitate the discovery of *cis*‐regulatory interactions [25–28] or regulatory networks [25, 29–32] specific to subpopulations. In addition to unraveling regulatory mechanisms in healthy cells, integrative multimodal analyses have the potential to unveil regulatory signatures [28] unique to cancer and provide insights into cancer evolution [33]. Ultimately, integrative analyses enable us to comprehend the interactions that occur within and between different molecular layers and their impact on gene expression.” was incorrect. This should have read: “Integrated multimodal analyses enable the identification of novel cell subtypes, subpopulation‐specific *cis*‐regulatory interactions and regulatory networks [16, 25–33], as well as cancer‐specific regulatory signatures [29] and evolutionary insights [34]. Ultimately, these approaches reveal inter‐ and intra‐layer molecular interactions and their effects on gene expression [16].”

(4) In paragraph 1 of the “2 Statistical challenges associated with single‐cell paired and unpaired multimodal data integration” section, the text “Due to the wide range of experimental assays, modalities, and biological questions the task of integrating multimodal data is not a singular well‐defined task. We can distinguish between two distinct integration tasks: integrating data from multiple modalities that originate from the same cell (referred to as paired data), and integrating data from different cells that belong to similar but nonidentical cell populations (referred to as unpaired data).” was incorrect. This should have read: “Single‐cell multimodal integration is best viewed as a design‐dependent family of problems rather than a single recipe because assay panels, protocols, and study aims differ widely. Following the pairing‐based taxonomy introduced by Ref. [16], we structure the landscape by whether measurements from different modalities are co‐assayed for the same cell (paired) or collected on different cells from related populations (unpaired).”

(5) In paragraph 2 of the “2 Statistical challenges associated with single‐cell paired and unpaired multimodal data integration” section, the text “In the case of paired data, where cell‐to‐cell correspondences are known, the primary objective is to improve the identification of cell states. On the other hand, unpaired data integration primarily focuses on identifying cell‐to‐cell correspondences. These fundamental differences in integration tasks have led to the development of tools that are generally designed for either paired or unpaired data integration.” was incorrect. This should have read: “Following Ref. [16], we distinguish two regimes. When modalities are co‐assayed for the same cell (paired), methods exploit within‐cell links to refine state delineation and characterize cross‐modal dependence. When measurements come from different cells (unpaired), integration reduces to estimating probabilistic correspondences via distribution/embedding alignment and anchor discovery, with uncertainty assessed under potential composition shifts. These contrasts motivate distinct toolkits for each regime.”

(6) In paragraph 6 of the “2 Statistical challenges associated with single‐cell paired and unpaired multimodal data integration” section, the text “Biological factors variation between modalities: Variations can arise due to chromatin priming, where changes in chromatin accessibility precede gene expression changes or convergence of lineages.” was incorrect. This should have read: “Biological factors variation between modalities: Asynchronous regulatory dynamics, post‐transcriptional control and turnover, cell‐cycle stage, and microenvironmental cues may decouple signals across assays [16].”

(7) In paragraph 1 of “4 Model Characteristics” section, the text “The results are shown in Table 4 for paired data methods” was incorrect. This should have read: “The results are shown in Table 4 [16] for paired data methods.”

(8) In paragraph 2 of the “6 Conclusion” section, the text “For instance, many integration methods fail to account for dataset‐specific cell populations that may arise from technical variations between assays or biological distinctions across modalities. In the case of CCA‐based approaches [85, 86], the inherent assumption of CCA—that a linear relationship exists between variables—may be invalidated for gene expression and chromatin accessibility profiles, given the intricate nonlinear processes governing gene expression regulation. Methods relying on similarity kernels, which aim to discern a shared underlying manifold, might prove more suitable. However, the selection of a similarity kernel in these methods is intricate and dependent on the modality. Notably, the choice of the similarity kernel and the features used for its computation (e.g., genes or genomic regions) will impact the biological signals retrieved, akin to decisions regarding other pre‐processing steps.” was incorrect. This should have read: “Integration strategies for paired and unpaired single‐cell data face distinct challenges: harmonizing modalities for the former, and resolving fine‐grained cellular states for the latter [85, 86]. Benchmarking reveals that performance varies significantly with data type and biological context. For paired data, methods such as scAI and DCCA excel in simple cellular compositions but struggle with highly heterogeneous populations, highlighting a need for improved resolution of subtle cell states. For unpaired data, Seurat v3, Liger, and scJoint effectively mix modalities while preserving biological separations. Deep learning‐based approaches outperform others on metrics such as ARI and NMI, demonstrating the advantage of graph neural networks in capturing shared signals. In contrast, methods such as VIPCCA lag in cluster compactness, underscoring fundamental differences in latent space construction among methods. Users should therefore select methods based on their data type and analytical goals, with tools such as Cobolt and multiVI available for both scenarios.”

(9) In paragraph 3 of the “6 Conclusion” section, the text “The increasing availability of atlas‐sized single‐cell omics datasets necessitates the development of methods capable of integrating expression and accessibility profiles for thousands to millions of cells [87, 88]. As the search space expands, finding appropriate cell‐to‐cell matches becomes more challenging. To address this, a constrained integration strategy has been proposed, wherein the alignment problem is divided into separate integration tasks for groups of cells that share a coarse‐grained cell identity (e.g., T cells and non‐T cells).” was incorrect. This should have read: “A critical consideration is the use of the gene activity matrix, employed by methods such as scMVAE and VIPCCA to integrate scATAC‐seq and scRNA‐seq data [87, 88]. Although this reduces modality differences, it risks information loss or bias, underscoring the need for better strategies to utilize chromatin accessibility data. Additionally, the growth of spatial transcriptomics has spurred interest in integrating these data with scRNA‐seq. Although beyond this review’s scope, tools such as Seurat and Liger already enable such analyses, opening avenues to explore cellular spatial organization and communication.”

(10) In paragraph 4 of the “6 Conclusion” section, the text “Moreover, most existing methods are not designed to handle the scale of thousands to millions of expression and accessibility profiles, often due to excessive runtime or memory requirements. Therefore, efforts are required to scale up existing approaches (as demonstrated with Seurat v3 in ArchR) and to develop inherently scalable methods, such as deep learning‐based approaches. It is crucial that these methods maintain scalability even when hyperparameter optimization is necessary. Hyperparameter optimization poses another bottleneck, particularly due to the reliance on labeled data. Paired multiomics data can contribute to addressing this challenge by providing labels for semi‐supervision and informing hyperparameter optimization strategies suitable for unpaired multiomics data integration tasks. We believe that, in the years to come, more versatile and powerful computational methods will efficiently and accurately harmonize a wide range of data and accelerate life sciences research [89–91].” was incorrect. This should have read: “In summary, single‐cell multi‐omics data integration is a dynamic field with great potential to illuminate cellular biology and disease [89–91]. As data scales increase, the demand for robust methods grows. We anticipate that more versatile and powerful computational tools will emerge to efficiently harmonize diverse data types, thereby accelerating discovery across the life sciences.”

(11) In the “References” section, a change in the reference sequence has been made: the former reference [73] is now listed as [16]. Consequently, all references from [16] to [72] have been incremented by one.

We apologize for above errors.

